# Knowledge, attitude, and practice toward medication use among pregnant women attending Mansoura university hospital antenatal care clinics: an analytic cross-sectional study

**DOI:** 10.1186/s12884-025-08430-1

**Published:** 2026-01-13

**Authors:** Tayseer Metwally, Mariam Orma, Nada Elbostany, Yahia Ali, Noha M. Abu Bakr Elsaid, Hebatalla Aly

**Affiliations:** 1 Faculty of Medicine, Suez University, Suez, Egypt; 2 Faculty of Medicine, Suez Canal University, Ismailia, Egypt

**Keywords:** Attitude, Knowledge, Medication, Practice, Pregnancy

## Abstract

**Background:**

Medication use during pregnancy is so prevalent worldwide. Self-medication may have drawbacks for maternal and fetal health. In Egypt, there are scarce studies assessing the knowledge, attitude, and practice (KAP) of pregnant women regarding the use of non-prescribed medications during pregnancy.

**Methods:**

An analytic cross-sectional study was implemented using a structured interview to assess the KAP of pregnant women attending the antenatal care clinics at Mansoura University Hospital toward medication use during pregnancy.

**Results:**

Out of 387 respondents. 30.5% of pregnant females used self-medication. Most participants demonstrated inadequate levels of knowledge, with 43% of participants classified as having poor knowledge, 39% fair knowledge, and 18% having good knowledge. Regarding Attitude, 94.6% of participants had a positive attitude, and 20% demonstrated good practice of medication use during pregnancy.

Binary logistic regression analysis showed that participants’ attitudes and practices are significantly associated with using non-prescribed medications. Participants with negative attitudes were 8.5 times more likely to use non-prescribed medications compared to those with positive attitudes. Also, participants with poor practice were 1.6 times more likely to use non-prescribed medications compared to those with moderate or good practice.

**Conclusions:**

The study revealed inadequate levels of knowledge, and high level of poor practice among pregnant women that is associated with increased use of self- medication despite their positive attitude towards risks of medication use during pregnancy, therefore we recommend the implementation of an educational program to improve awareness among pregnant women about the importance of using medications only under medical supervision to enhance their practices.

## Background

Medication use during pregnancy is prevalent up to 44–99% [1–5]. Globally, it has become obvious that the use of medications, either with or without a physician's prescription, among pregnant women has increased in the past years [6–9]. Non-prescribed medication is a serious and expanding global public health concern. The rate of self-medication varies greatly by area, from 38.5% to 92%, suggesting that a sizable section of the world's population consumes pharmaceuticals without seeking appropriate medical advice. In underdeveloped nations, about 80% of drugs are bought without a prescription [10].

According to the World Health Organization (WHO), self-medication (SM) or non-prescribed medication is the practice of using medication to cure illnesses or disorders that one has self-diagnosed. Using over-the-counter drugs (OTCs), medication, or exchanging pharmaceuticals with friends or family are only two examples of how people may use medications without a doctor's supervision [11, 12]. Pregnant women frequently suffer from headaches, nausea, vomiting, and constipation, which often prompts them to self-medicate with herbal remedies or over-the-counter (OTC) medications [13]. However, self-medication without expert advice puts the health of the mother and fetus in danger [14]. Few over-the-counter medications are categorized as fully safe (Categories A and B) by the U.S. Food and Drug Administration (FDA), which divides medications into five pregnancy risk groups (A, B, C, D, and X) [15]. It was found that self-medication during pregnancy is 1.5 times more common than prescribed medications [16].

Approximately 3–5% of live births are complicated by a congenital disability each year. Susceptibility to teratogenic agents varies with the developmental stage at the time of exposure. The ultimate manifestations of abnormal development are death, malformation, growth retardation, and functional disorder [17].

Non-prescription medicine use during pregnancy varies by country, with rates of 43.9% in Italy [6], 44.6% in Ethiopia [18], 58.19% in KSA [19], and 73.3% in Port Said and Damietta, Egypt [13]. Pregnant women in India have a positive attitude (91.33%) and good knowledge (75.33%) about using medications safely, according to a previous study [12], but practice is relatively low (35%). However, in Egypt, 47.7% of participants knew enough about medication use during pregnancy. Women who were younger than thirty years old, illiterate, housewives, primigravida, and had a history of abortions were among the characteristics that seemed to influence their awareness of medication use during pregnancy. Another previous study reported that Pregnant women who took medicine were somewhat older (*P* = 0.027) and more likely to reside in an urban setting (*P* = 0.002). The most commonly utilized class was analgesics (70.3%). The main reasons for Self-medication were the moderate nature of the illnesses, the availability of an existing prescription, and advice from pharmacists [19].

A recent systematic review and meta-analysis reported that the overall prevalence of non-prescribed medication use among pregnant women varies from 9.67% to 36.71% across different regions in Ethiopia. The most frequently used non-prescribed drug by pregnant mothers was paracetamol [20].

The use of medication during pregnancy is a complex issue that requires careful consideration to avoid potential harm to the developing fetus. Although a few studies in Egypt have assessed the KAP of pregnant women regarding medication use, there are very few studies reported on the use of medications during pregnancy. This study aimed to evaluate the KAP (Knowledge, Attitude, and Practice) of pregnant women regarding medication use during pregnancy.

## Methods

### Type of the study

An analytic cross-sectional study.

### Sample

#### Sampling

Systematic random sample (every second patient entering the clinic).

#### Sample size

The sample size was estimated according to the following equation:

$$n\;=\left[\frac{{\mathrm Z}_{\propto/2}}{\mathrm E}\right]^2\ast\mathrm P\left(1-\mathrm P\right)$$

n = required sample size,

Zα/2 = 1.96 (The critical value that divides the central 95% of the Z distribution from the 5% in the tail)

*p* = the prevalence of good knowledge level (0.477) [21]

E = the margin of error (= width of confidence interval) = 0.05

So, n = 383

10% was added for a non-response rate of 38

The final sample size will be 421 participants

### Study setting

Outpatient Clinic for Antenatal Care (ANC), Mansoura University Hospital. Egypt.

### Study population

#### Inclusion criteria

The study includes all pregnant women, primigravida or multigravida, in any trimester of pregnancy, with or without chronic disease, who attended the outpatient clinic for antenatal care, at Mansoura University Hospital, antenatal care outpatient clinics.

#### Exclusion criteria

Non-Egyptian women and pregnant women who were admitted to the inpatient unit.

## Methods

An interviewer-administered questionnaire was used with pregnant women attending the ANC clinics at Mansoura University Hospital. A predesigned 24-item questionnaire, previously tested, validated, and reliable, had been used to assess the KAP toward medication use during pregnancy [6]. The questionnaire underwent a bidirectional translation and Arabic version was evaluated by a panel of 3 experts (obstetric & gynaecology, family medicine. and public health consultants).

Data collection had been done in two months, five days a week, at Mansoura University Hospital, antenatal care outpatient clinics, by the researchers in a private waiting room. The length of the interview was approximately 20 min. The participants were assured that their data was kept confidential because no names were recorded. They did not receive any compensation for participating.

### The questionnaire consisted of 5 sections

*The first section* explored socio-demographics (i.e., age, marital status, education level, employment status) and medical data (i.e., medical history, gravidity, parity, gestational age, number of pregnancies).

*The second section* had been used to measure the level of knowledge about specific aspects of medication use during pregnancy, including the possibility of harm to the unborn (e.g., fetal growth retardation, intrauterine death, malformations) and to their health, and the potential risk regarding the use of non-prescribed medications. Total knowledge score was summed up and graded according to Bloom’s rule as follows: < 50% poor knowledge, 50–80 fair knowledge, and > 80% good knowledge. Although the classic Bloom’s cutoffs are often reported as 80–100% (good), 60–79% (moderate), and < 60% (poor) [22], but we adopted a modified scheme (< 50% poor; 50–80% fair; > 80% good) to improve discrimination in our sample, as used by other recent KAP studies [23, 24].

*The third section* had been used to assess whether respondents accept the idea of using medications while pregnant without the prescription of a physician. Participants are also asked about the reasons for their attitude. Participants who answered yes with different reasons were considered to have a negative attitude, while participants who answered no with different reasons were considered to have a positive attitude.

*The fourth section* had been used to evaluate the practice, by asking if the women have used medications, not including vitamins, mineral supplements, and herbal treatment (i.e., health problem for the use, compliance to dose regimens, duration of treatment), with or without a prescription during their current pregnancy. Total practice score was summed up and graded according to modified Bloom’s rule as follows: < 50% poor practice, 50–80 moderate practice, and > 80% good practice.

*The fifth section* had been used to evaluate the sources of information on medications used during pregnancy and the interest in learning more.

### Statistical analysis

Data were analyzed by the statistical program SPSS version 26. Qualitative data expressed in numbers and percentages. Chi-square test used for comparison between groups. Significance level defined at *P*-value < 0.05, and a binary logistic regression analysis was used to identify factors associated with the KAP levels of pregnant women, and the results were displayed in a suitable form of tables and figures.

## Results

(Table 1) shows that a total of 387 pregnant women at Mansoura University Hospital outpatient clinics participated in this study. The mean age of participants was 27 ± 6 years, most participants (91.5%) were housewives, (53.5%) had secondary education, 70.5% multigravida, 33.3% had experienced previous abortions, and 71.7% were in their 3rd trimester.

**Table 1:** Socio-demographic characteristics of study participants (*N* = 387)

Socio-demographic characteristics of study population	N	%
Age in years		
18- 24	162	41.9
25- 34	167	43.2
≥ 35	58	14.9
Mean ± SD	27 ± 6	
Education		
Illiterate	38	9.8
Read and Write	8	2.1
Primary	49	12.7
Secondary	207	53.5
University	85	22
Occupation		
Employee	33	29.5
Housewife	354	70.5
Gravidity		
Primigravida	114	29.5
Multigravida	273	70.5
Number of previous abortions		
No previous abortion	258	66.7
one abortion	69	17.8
≥ 2 abortion	60	15.5
Gestational age		
1 st trimester	29	7.5
2nd trimester	83	21.4
3rd trimester	275	71.1

**Fig. 1:**
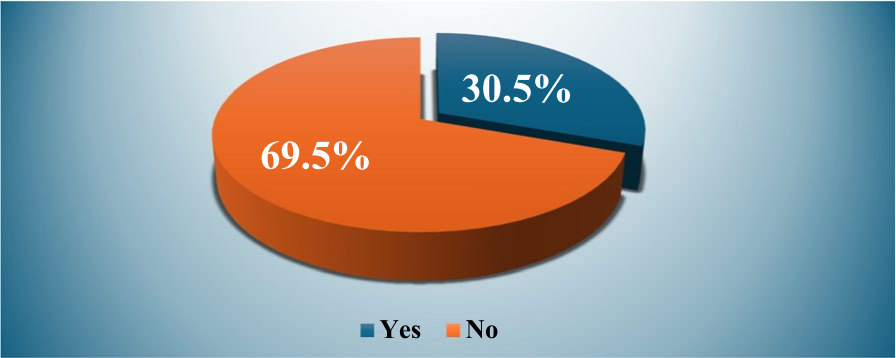
Frequency of using medications without prescription among study population (*N* = 387)

Regarding the use of medication without a prescription, Fig. 1 shows that 30.5% of study participants used medication during pregnancy without a prescription.

**Fig. 2:**
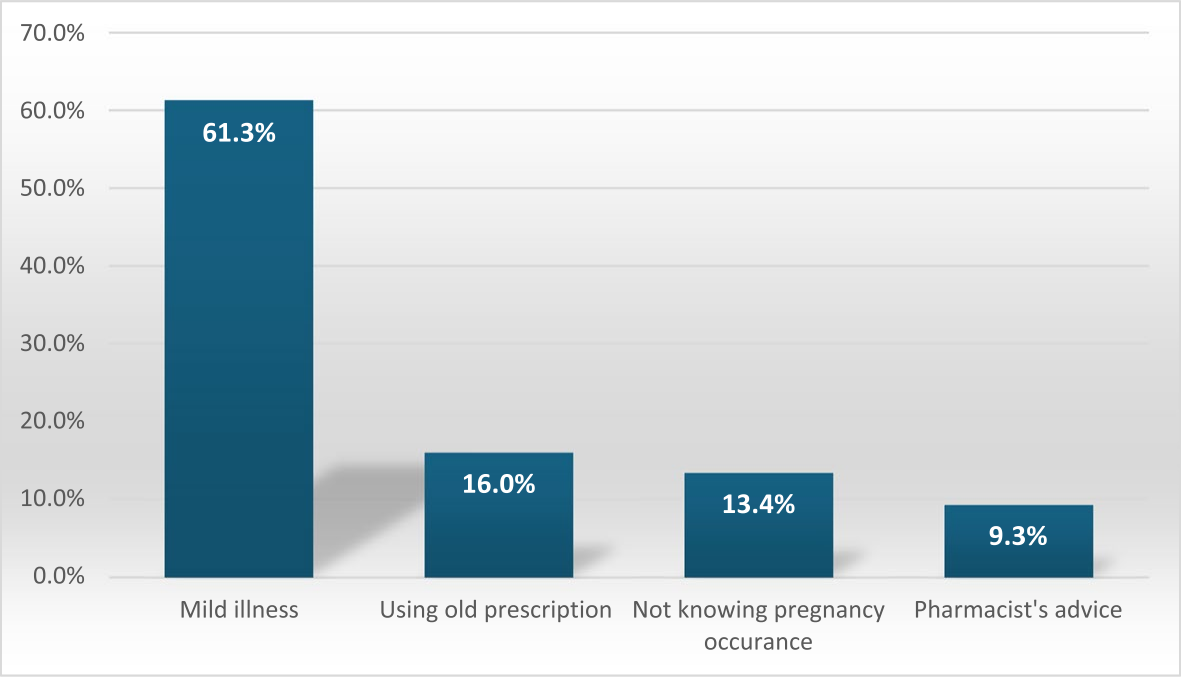
Participants stated their causes of using medication without prescription (*N* = 119)

Participants were asked about using medications without a prescription. About 61.3% of respondents perceived their illness as mild, which did not require a visit to the doctor, 16% relied on an old prescription, 13.4% were unaware of their pregnancy, and 9.3% based their decision on the pharmacist’s advice (Fig. 2).

Participants were asked about the effects of medication use during pregnancy on fetal health. 54.3% recognized the risk of fetal growth retardation, and 48.8% acknowledged the possibility of intrauterine death. Chromosomal abnormalities were identified as a potential consequence by 79.1%, and 53% believed that medication use could lead to visible malformations in subsequent ages. Regarding the effects of medication on maternal health, the risk of abortion, uterine contractions, bleeding, and postpartum depression was acknowledged by 66.7%, 67.2%, 54.3%, and 33.6% respectively. Participants were asked whether medications should be used during pregnancy or not; 94% stated that the physician’s advice is necessary, and 89.7% knew that they are potentially harmful (Table 2).

**Table 2:** Knowledge of study population regarding medication use during pregnancy (*N* = 387)

	Yes N. (%)	No N. (%)	I don’t know N. (%)
Effects of medication use during pregnancy on the fetus and newborn			
•Fetal growth retardation	210 (54.3)	29 (7.5)	148 (38.2)
• Intrauterine death	189 (48.8)	29 (7.5)	169 (43.7)
• Chromosomal abnormalities	306 (79.1)	12 (3.1)	69 (17.8)
• Visible malformations in subsequent ages	205 (53)	45 (11.6)	137 (35.4)
Effects of medication use during pregnancy on the pregnant			
• Abortion	258 (66.7)	21 (5.4)	108 (27.9)
• Uterine contraction	260 (67.2)	13 (3.4)	114 (29.5)
• Bleeding	210 (54.3)	29 (7.5)	148 (38.2)
• Postpartum depression	130 (33.6)	55 (14.2)	202 (52.2)
• Loss of memory	56 (14.5)	83 (21.4)	248 (64.1)
Non-prescribed medications can be used during pregnancy			
• No, the physician’s advice is necessary	364 (94.1)	23 (5.9)	
• No, they are potentially harmful	347 (89.7)	30 (10.3)	
• Yes, the pharmacist must provide all necessary advice and information	20 (5.2)	367 (94.8)	
• Yes, they have less side effects	2 (0.5)	385 (99.5)	

**Fig. 3:**
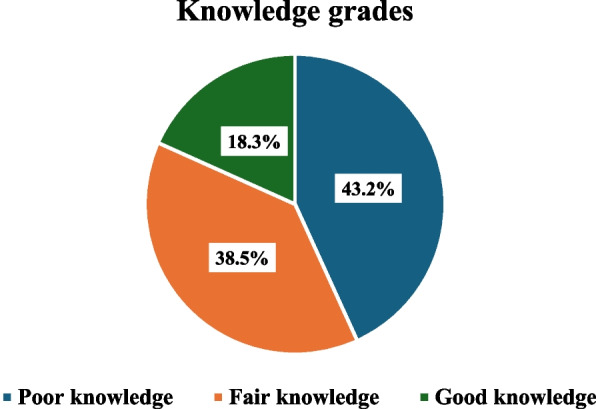
Distribution of study participants according to their knowledge scores regarding medication use during pregnancy (*N* = 387)

The overall knowledge level was based on knowing medications' effects on the mother and fetus, and it indicated that most participants demonstrated inadequate levels of knowledge, with 43% of participants classified as having poor knowledge, 39% fair knowledge, and just 18% having good knowledge (Fig. 3).

**Fig. 4:**
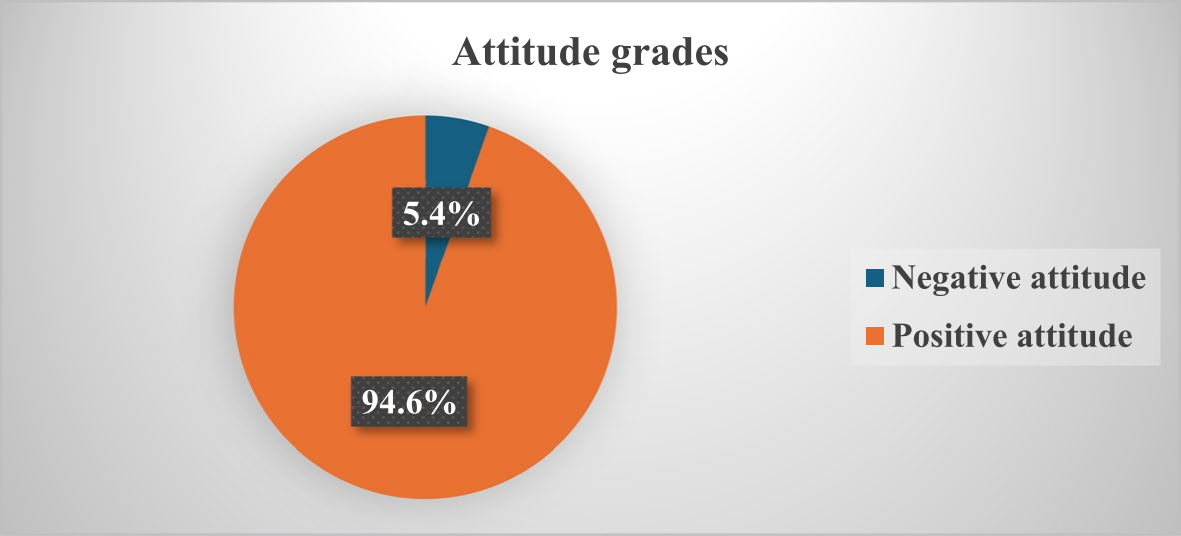
Attitude grades among study participants (*N* = 387)

Table 3 shows participants’ attitudes towards the use of non-prescribed medications during pregnancy. Participants who stated yes chose from different reasons and represent a negative attitude, and were 5.4% of participants. On the other hand, participants who stated no choice for various reasons and represent a positive attitude were 94.6% of participants (Fig. 4).

**Table 3:** Study population attitude regarding medication use during pregnancy (*N* = 387)

Non prescribed medications should be used during pregnancy	N.	%
Yes (negative attitude)	2	0.5
The health problem is not serious‎	0	0
I have an old medical prescription‎	3	0.8
An emergency ‎‎	0	0
I am traveling ‎	7	1.8
‎The physician is not available‎	19	4.9
‎Pharmacist’s advice ‎	0	0
Friends and relatives’ advice‎	0	0
The physician does not prescribe it‎	0	0
The physician is not easily accessible (times and distance)‎	6	1.6
Knowledge about the side effects‎	1	0.3
Previous experience of using medications in pregnancy without physician’s ‎prescription	4	1
The health problem is not serious‎	2	0.5
Total participants with negative attitude	21	5.4
No (positive attitude)		
I am afraid of side effects	336	86.8
I will wait for a medical consultation	346	89.4
I use only natural products	191	49.4
I am concerned about the risk for the unborn baby	351	90.7
I am concerned about the risk for my health	330	85.3
I have already used it, and I have had problems	17	4.4
Total participants with positive attitude	366	94.6

The results of participants’ practice regarding medication use during pregnancy in Table 4 show that 24.3% will consult the doctor first about whether medication used for a chronic disease should be modified or not, 13,4% didn’t know, and 45.7% say that the pregnant woman should know whether or not to use medication. About 86.8% didn’t know that medication was used according trimester of pregnancy, 55.6% read the drug leaflet to know its safety in pregnancy, and 61.5% needed to know more about the medication in pregnancy.

**Table 4:** Study population practice regarding medication use during pregnancy (*N* = 387)

Practice questions	N	%
The medications used for chronic disease should be modified during pregnancy?		
No	40	10.3%
A woman should know whether or not to use a medication	177	45.7%
The use must be suspended/stopped because it is harmful for the unborn baby	23	5.9%
The use should be suspended/stopped because it is harmful for the woman	1	.3%
I will consult a doctor first	94	24.3%
I didn’t know	52	13.4%
Should the medication you are taking during pregnancy, whether vitamins or medications for chronic diseases, be changed during pregnancy?		
No, never	80	20.2%
Only in the first trimester	7	1.8%
Only in the second trimester	9	2.3%
Only in the third trimester	9	2.3%
At any time of pregnancy	90	23.3%
I Will consult a doctor first	141	36.4%
I didn’t know	51	13.2%
Do you read the drug leaflet to know if the drug is safe for pregnancy or not?		
Yes	215	55.6%
No	172	44.4%
Do you feel you need to gather more information about the use of medications in pregnancy?		
Yes	238	61.5%
No	139	38%

**Fig. 5:**
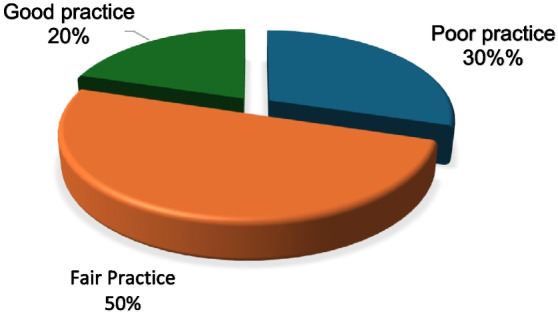
Distribution of study participants according to their practice scores regarding medication use during pregnancy (*N* = 387)

Figure 5 shows the distribution of participants according to their total practice score. Half of the participants exhibited fair practice, while 30% showed poor practice, and only 20% showed good practice.

Table 5 shows that no statistically significant association was observed between sociodemographic characteristics and level of knowledge, attitude, or practice regarding medication use during pregnancy, except for parity, as nulliparous participants showed fairer to good knowledge compared to multiparous and this was statistically significant.

**Table 5:** Association between the level of knowledge and practice of pregnant women regarding medication use and socio-demographic characteristic (*N* = 387)

Characteristic	Knowledge		Attitude		Practice	
	Fair/good N (%)	Poor N (%)	Positive N (%)	Negative N (%)	Moderate/good N (%)	Poor N (%)
Age						
15–24	91 (56.2%)	71 (43.8%)	155 (95.7%)	7 (4.3%)	108 (66.7%)	54 (33.3%)
25–34	92 (55.4%)	74 (44.6%)	156 (93.4%)	11 (6.6%)	124 (74.3%)	43 (25.7%)
35–45	36 (62.1%)	22 (37.9%)	55 (94.8%)	3 (5.2%)	40 (69%)	18 (31%)
* p-*value	0.667		0.660		0.313	
Employment						
Employee	22 (68.8%)	10 (31.3%)	29 (87.9%)	4 (12.1%)	23 (69.7%)	10 (30.3%)
Housewife	197 (55.6%)	157 (44.4%)	337 (95.2%)	17 (4.8%)	249 (70.3%)	105 (29.7%)
*p*-value	0.152		0.076		0.938	
Medical problems						
Yes	89 (54.9%)	73 (45.1%)	154 (95.1%)	8 (4.9%)	123 (75.9%)	39 (24.1%)
No	130 (58%)	94 (42%)	212 (94.2%)	13 (5.8%)	149 (66.2%)	76 (33.8%)
*p-*value	0.544		0.719		0.039	
Gravidity						
Primigravida	72 (64.9%)	39 (35.1%)	3 (2.7%)	108 (97.3%)	75 (67.6%)	36 (32.4%)
Multigravida	147 (53.5%)	128 (46.5%)	18 (6.5%)	258 (93.5%)	197 (71.4%)	79 (28.6%)
*p-*value	0.041^*^		0.134		0.458	
Abortion						
Yes	80 (62%)	49 (38%)	124 (96.1%)	5 (3.9%)	95 (73.6%)	34 (26.4%)
No	139 (54.1%)	118 (45.9%)	242 (93.8%)	16 (6.2%)	177 (68.6%)	81 (31.4%)
*p-*value	0.138		0.341		0.307	
Trimester of pregnancy						
1 st Trimester	19 (65.5%)	10 (34.5%)	28 (96.6%)	1 (3.4%)	20 (69%)	9 (31%)
2nd Trimester	51 (61.4%)	32 (38.6%)	81 (97.6%)	2 (2.4%)	61 (73.5%)	22 (26.5%)
3rd Trimester	149 (54.4%)	125 (45.6%)	257 (93.5%)	18 (6.5%)	191 (69.5%)	84 (30.5%)
*p-*value	0.320		0.307		0.769	

Chi square test was used;

^*^Statistically significant at *p* < 0.05

The difference between knowledge, attitude, and practice grades among participants who use and those who don’t use non-prescribed medications is shown in Table 6. Negative attitude, moderate and poor practice grades were associated with using non-prescribed medications, and these were statistically significant.

**Table 6:** The difference between knowledge, attitude and practice grades among participants who use and those who don’t use non- prescribed medications (*N* = 387)

KAP grade		Use of non-prescribed medications		*p*-value
		Yes (*n* = 118)	No (*n* = 267)	
Knowledge grade	Poor	54 (32.3%)	113 (67.7%)	0.446^a^
	Moderate	47 (31.5%)	102 (68.5%)	
	Good	17 (24.3%)	53 (75.7%)	
Attitude grade	Negative	16 (76.2%)	5 (23.8%)	< 0.001^a*^
	Positive	102 (27.9%)	264 (72.1%)	
Practice grade	Poor	46 (40%)	69 (60%)	0.012^a*^
	Moderate	56 (28.9%)	138 (71.1%)	
	Good	16 (20.5%)	62 (79.5%)	

^a^Chi square test;

^*^Statistically significant at *p* < 0.05

As shown in Table 7, participants’ attitudes and practices are significantly associated with using non-prescribed medications. Participants with negative attitudes were 8.5 times more likely to use non-prescribed medications compared to those with positive attitudes. Also, participants with poor practice were 1.6 times more likely to use non-prescribed medications compared to those with moderate or good practice.

**Table 7:** Logistic regression analysis of non-prescribed medications use and each of attitude and practice grades

Covariates	β	*p-*value	OR (95% CI)
Attitude grades	2.142	< 0.001^*^	8.516 (3.008–24.112)
Practice grades	0.495	0.003^*^	1.641 (1.179–2.284)
Constant	2.175	0.014^*^	0.117
Model χ^2^ = 28.715		< 0.001^*^	

^*^Statistically significant at *p* < 0.05

## Discussion

This study provides valuable insights about the knowledge, attitude, and practice of pregnant women regarding non-prescribed medication use during pregnancy in MUH, Egypt. Among study participants, 30.5% of pregnant women used drugs without a prescription, which is considered low in comparison to other studies conducted in Egypt. The percentage of pregnant women who used drugs without a prescription was 73.3% in Port Said [13], and 82.5% in Menoufia [25]. The socio-demographic characteristics of the recruited sample could explain this result. The majority of study participants were housewives, which makes it easier for them to find the time and opportunity to monitor their pregnancies at Mansoura University's government health facilities. This contrasts with working women, particularly during working hours. Additionally, this result is consistent with Khalaf et al.'s findings [26]. Moreover, above one-third (33.3%) of our study participants had a history of abortion, which may contribute to decreasing the prevalence of non-prescribed medication use. According to a previous study, Women who did not have a history of abortion had a 2.85-fold higher odds ratio of self-medication during pregnancy than women who had at least one abortion. Aborted women appear to be less inclined to self-medicate because they are more sensitive to their pregnancy status and are less likely to self-medicate [27]. Furthermore, over half of the women in the study had only completed secondary school, which is consistent with the findings of a prior study that found that pregnant women with academic and high school education had the highest prevalence of self-medication because they could obtain adequate information from drug brochures [28]. One of the basic components of non-prescription medication is herbal medicine or home remedies that are used on one's own initiative or at the recommendation of another person without a medical prescription [29]. While the study evaluated the use of non-prescription medications during pregnancy, it did not include any detailed inquiries about them.

Also, this outcome is less than observed in eastern Mediterranean and African countries like the Emirates [30], Ethiopia [18], Nigeria [31], and Saudi Arabia [32], where the prevalence was 40%, 44.6%, 72.4%, and 73.8% respectively. On the other hand, the reported prevalence of non-prescribed medication is higher than observed in developed countries such as Texas [33] and in Holland [34], where it was 23% and 12.5% respectively. The prevalence of self-medication among pregnant women was 44.50% worldwide between January 2011 and December 2021, with a range of 2.61% to 85%, according to a recent systematic and meta-analysis study [35].

This discrepancy in the prevalence of use of non-prescribed medications between the current study and other studies is due to the fact that the prevalence of self-medication varies greatly between developed and developing countries due to differences in sociocultural context and economic status [36].

The most common reasons for self-medication among pregnant women in the current study were the perception that the illness was not serious (61.3%), reliance on an old prescription (16%), respectively. Similar to an earlier study conducted in Ethiopia found that the reported reasons for self-medicating were the easy underestimating the severity of their illness, and prior successful self-medication [37] in line with previous studies in Mansoura [38], Ethiopia [18], and Southern Italy [6], where 86.7%, 40.3%, and 48.2% of participants, respectively, thought their disease was minor.

This study found that the most common medications used without prescription were acetaminophen/Panadol (64%), antibiotics (8%), and NSAIDs‏ (8%). In accordance with a recent study conducted in Saudi revealed that the most used over-the-counter (OTC) medications were analgesics/antipyretics (92.4%) [39]. Contrary to a previous study reported that the most prevalent supplements taken by pregnant women were calcium (28.6%), iron (35.1%), and folic acid (36%) [30].

According to study participants' awareness of the risks associated with using medications during pregnancy, 79.7% of women acknowledged that there is a chance of chromosomal abnormalities in the fetus; this number is consistent with a study conducted in Southern Italy. It revealed that 81.1% were aware that using drugs during pregnancy could harm the fetus (e.g., fetal growth retardation, intrauterine mortality, deformities) [6]. Additionally, 54.3% of participants in the current study reported fetal development retardation as a risk, and 48.8% reported intrauterine death. In accordance with a prior study conducted in Mansoura, 70% of respondents acknowledged the risk of delayed growth associated with drugs, whereas 9% recognized the potential for stillbirth [38]. Contrary to a Saudi Arabian study, most women who had previously given birth to an abnormal child did not believe that drug usage during pregnancy was the cause of this [32].

Regarding the current maternal health hazards from non-prescribed medication use during pregnancy, 66.7% recognized the risk of abortion. This outcome is consistent with the results in a previous study in Mansoura [38]. The current study reported that a higher percentage of pregnant women were aware that certain medications may cause vaginal bleeding (45.3%) compared to a previous study conducted three years earlier in Mansoura (7.8%). This difference may be due to improved public awareness over time, the inclusion of participants from a university hospital with better access to health education, and possible variations in sample characteristics or data collection methods.

 Concerning the study's findings on pregnant women's attitudes toward self-medication during pregnancy, 90.7% were worried about the risk to the fetus, 89.4% would wait for a doctor's appointment, and 86.8% were terrified of adverse effects. Comparatively, a prior study conducted in Southern Italy revealed that 39% were worried about side effects, 77.8% would wait for their obstetrician's recommendation, and 52.8% were worried about the risk to the unborn child [6].

Regarding pregnant women's practice, 55.6% reported reading the leaflet. This finding is lower than in the Emirates and Saudi Arabia, which were 70% and 78% respectively [30, 32]. Only 18% of participants were found to be well informed, while 38.5% had a fair level of knowledge on the effects of self-medication on the mother and fetus. Just 20% of women demonstrated good practice, while 30% showed poor practice. This suggested that the majority of study participants showed insufficient practice and a low knowledge level. A recent study in Saudi Arabia revealed that only 37.5% of women showed good awareness of the risks of self-medication use, while 54.8% showed a moderate level of awareness [39]. The characteristics of the current study population may be the cause of these findings, which reflect the moderate level of education of the studied participants.

No statistically significant association was observed between socio-demographic characteristics and level of knowledge or practice regarding medication use during pregnancy, except for gravidity status in the present study. This indicates that the level of knowledge and practice didn’t differ among different socio-demographic characteristics. Contrary to the current study, a prior Ethiopian study found that younger age, living in a rural area, and prior experience with self-medication were independent factors linked to expectant women's use of self-medication [18]. The current study found that Primigravida were more knowledgeable about non-prescribed medication use, unlike other previous studies in Ethiopia, Iran, and Uganda, which reported higher self-medication use among primigravida than multiparas [37, 40, 41]. There is no scientific explanation for this in scientific literature except that Primigravida is more sensitive and afraid of the possible consequences to the fetus, which may make them less prone to self-medication.

According to the current study, non-prescribed medication use and positive attitude were significantly associated, while no significant relationship between knowledge level and self-medication use. Similarly, Knowledge did not significantly reduce the use of self-medication, according to a prior study in Egypt (an OR: 1.15; CI: [0.90–1.48], *p*-value = 0.268). Positive attitudes were associated with considerably lower chances of self-medication (aOR: 0.44; CI: [0.36–0.55], *p*-value < 0.001). These results demonstrate that the usage of self-medication is not solely a function of knowledge level [42]. This indicates that simply knowing the risks does not necessarily prevent pregnant women from self-medicating.

In contrast, a prior study conducted in Italy found a significant association (*P* = 0.003) between the use of drugs during pregnancy and accurate knowledge of the potential harm to the mothers [6]. This study found that women who believed that chronic disease medications should be modified during pregnancy were significantly more likely to self-medicate (OR = 1.857, *p* = 0.007). This suggests a possible misapplication of that belief, potentially leading to self-medication without medical supervision. Interestingly, those who knew that physician advice is necessary were over 8 times more likely to have self-medication (OR = 8.317, *p* = 0.003). This may reflect a gap between awareness and action (they know advice is needed, but still self-medicate).

The study findings can inform policy on regulation OTC drugs. Regarding clinical practice, effective counseling strategies during ANC to enhance pregnant women's awareness regarding medication practices during pregnancy. Encouraging pregnant women with chronic diseases to follow up regularly with healthcare providers and avoid self-medication or altering their treatment without professional guidance. Establish comprehensive guidelines with clear classification of medications used during pregnancy, to assist healthcare providers in prescribing the safest and most effective treatment for pregnant women.

### Study limitations

Participants were selected from those who visited the outpatient clinic for antenatal care; pregnant women who were at home were not taken into account. Additionally, information about income, residence, and insurance type was overlooked. This study did not include information on the detailed types of self-medication, the sources of drugs, symptoms linked to the most common non-prescription use, and side effects of the used drugs among pregnant women. Practice questions didn’t include asking about vitamins, mineral supplements, and herbal treatment. The study is cross-sectional; thus, it is impossible to conclude a cause-and-effect link from the data. Therefore, the study recommends prioritizing prospective observational studies that incorporate a wider population sample, and a complete medical history, including history of any clinical illness, the types, and sources of non-prescription medication.

## Data Availability

Data is available from the corresponding author upon request.

## Abbreviations

ANC: Antenatal care
FDA: Food and drug administration
KAP: Knowledge, attitude & practice
KSA: Kingdom of Saudi Arabia
MUH: Mansoura university hospital
OTC: Over the counter
SM: Self-medication
SPSS: Statistical package for the social sciences
